# “Today, I Say It’s Mine!”: Professional Identity Construction among Jewish and Arab School Counselors Coping with CSA Disclosure in Israel

**DOI:** 10.3390/bs14050424

**Published:** 2024-05-20

**Authors:** Dafna Zinn, Efrat Lusky-Weisrose, Jordan Shaibe, Laura I. Sigad, Dafna Tener

**Affiliations:** 1 Department of Inclusive Education, Faculty of Graduate Studies, Oranim Academic College of Education, Tivon 3600600, Israel; dafna_z@bialik.ort.org.il (D.Z.); jordan.shaibe@mail.huji.ac.il (J.S.); laura_s@oranim.ac.il (L.I.S.); 2 The Paul Baerwald School of Social Work and Social Welfare, Mount Scopus Campus, Hebrew University of Jerusalem, Jerusalem 9190500, Israel; dafna.tener@mail.huji.ac.il

**Keywords:** child sexual abuse (CSA), school counseling, socio-cultural contexts, professional identity construction

## Abstract

School counselors play a crucial role in preventing, disclosing, and intervening in child sexual abuse cases (CSA) and in maintaining safe and protected school environments. However, research on their experiences coping with CSA remains limited. The purpose of the present study was to describe and analyze the coping experiences of Israeli Jewish and Arab school counselors with CSA disclosure, particularly the consequences for their processes of professional identity construction (the ongoing process through which they develop and refine their sense of self in their profession). Semi-structured interviews were conducted with 21 Israeli Jewish and Arab school counselors working in elementary schools (grades 1–6) with significant experience in coping with CSA. Two themes surfaced, reflecting the counselors’ professional identity construction: (1) Counselors’ professional identity transformation following encounters with CSA among their students; (2) Integrating professional knowledge, attitudes, and engagement behaviors into professional identity. The findings describe a trajectory of transformation and professional development among the counselors, beginning with defining and refining their professional roles and followed by the integration of professional knowledge, attitudes, and behaviors into their professional routines. Arab counselors also highlighted specific sociocultural challenges within this process, relating to the gap between cultural values and role expectations. Implications for future research, policy, and practice are discussed.

## 1. Introduction

In the research on school counselors, a consensus has emerged that defining their role and promoting a sense of professional agency is essential for their autonomous and effective functioning [1]. How counselors’ professional identities are formed and how this impacts their competence in the workplace has been discussed both in a universal and local Israeli context (e.g., [2,3]). Identity formation is also an important consideration in the matter of counselors’ encounters with child sexual abuse (CSA), defined here as any type of physical or nonphysical sexual activity with a child under the age of 18. These acts occur in situations where the child cannot genuinely consent, emphasizing the child’s vulnerability and the power imbalance inherent in such cases [4]. CSA is a problem that affects children worldwide, frequently resulting in adverse consequences for their mental health and social functioning that can persist into adulthood [5].

School counselors, key figures in addressing violence both within and outside of school premises [6], play a crucial role in CSA prevention, detection, and reporting [7]. Counselors provide an in-school service that promotes the health and well-being of students and educators and seek to maintain a safe and protected environment in the educational setting [8,9]. As such, they are often the first recipients of CSA disclosure [10] and can facilitate disclosure by asking children directly about their experiences and providing active listening and support [11].

Since their role involves great responsibility, school counselors may be subject to immense emotional and professional pressure [10,12,13]. However, their experiences have been largely overlooked, specifically concerning their coping with CSA. The present study thus aims to examine the experiences and processes of professional identity construction of school counselors in Israel confronting CSA among their students, spotlighting the perceptions of both Jewish and Arab counselors.

### 1.1. Professional Identity Construction of School Counselors

The internalized psychological sense of being a professional, a phenomenon known as professional identity [14], encompasses a complex structure connecting a person’s motivations and competencies to their chosen professional role [15,16]. Through the development of moral principles and self-knowledge in a professional context [14], alongside the integration of skills and personal attitudes into their work, the individual begins to identify themselves with their professional role and to perceive themselves in the context of a professional community [17]. This process is active and dynamic and is a critical part of skill-based education [18]. The existing literature suggests that individuals with a strong professional identity can retain greater control over the outcomes and processes of their work when collaborating with others [3].

Past studies dealing with professional identity construction among school counselors identified it as the ongoing process through which these educators refined their sense of self in their professional roles [19,20,21]. As with other aspects of individual identity, the counselor’s professional identity is determined by the social context in which it is situated [16,22] and encompasses both interpersonal and intrapersonal aspects. Interpersonal dimensions include the counselor’s relationship to society and the professional community, as shaped by their cultural values, expectations, attitudes, knowledge, skills, and the beliefs of their profession. Intrapersonal dimensions include the evolution of one’s personal definition of counseling as reflection takes place and the locus of evaluation changes. New professionals tend to shift from external to internal evaluation; that is, from relying on experts to relying on their own history and training [23,24]. Through their experiences, reflections, and interactions [25,26], counselors align their work with their beliefs and expectations about student support, guidance, and advocacy [27]. However, the study of school counselors’ professional identity construction through their experiences with the CSA is limited, particularly in terms of their experiences in different sociocultural contexts.

### 1.2. The School Counselor’s Role in Contending with CSA

School counselors have a legal, ethical, and moral obligation to report suspected child abuse to the appropriate authorities [28]. In accordance with ASCA guidelines, they must demonstrate an understanding of child abuse problems, identify indicators of abuse, and provide prevention and intervention strategies [29]. Hence, they frequently serve as CSA prevention specialists and may advise or direct prevention programs targeted at students, school personnel, or parents [29]. A common feature of school-based child sexual abuse programs is teaching children to identify which “private parts” of their bodies should not be touched by others, as well as to identify when they feel uncomfortable when touched. Children are also taught how to assert their rights (e.g., to refuse unwanted touching); how to seek trusted adult support if they feel unsafe; and not to keep “bad secrets”. Additionally, they are encouraged not to blame themselves for their victimization [30]. Child abuse education lessons increase the student’s self-protection knowledge and skills and leads to earlier disclosure of abuse, preventing further abuse from occurring [31].

Likewise, counselors are qualified to train staff regarding definitions of child abuse as well as how to identify signs associated with sexually abused children [29]. They can also train teachers in how to appropriately respond to and report disclosures of CSA [32]. Counselors are key leadership figures [33,34] in addressing the crisis that CSA incidents can cause in school systems, which may affect student victims, their classmates, teachers, and parents, as well as the school’s organizational culture [35]. Counselors may be expected to act as case managers [9,36]; to provide individual and group interventions; to consult with authorities, parents, and professionals; to coordinate services with the school and community [36]; and to conduct follow-up over time [37].

### 1.3. The Profession of School Counseling in Israel

Since 2000, school counseling in Israel has transformed from a role fulfilled by teachers to a specialized profession requiring a master’s degree and two years of practical experience for licensure [38]. Broadly speaking, the counselor’s role is to promote the well-being of students and improve their functioning; however, this is a wide-ranging responsibility that spans issues such as developing a positive school climate, to improving teaching and learning process, to crisis intervention for individuals and groups [9].

Given this range, there is at present a lack of clarity regarding the role definition, boundaries, and demands of the counseling profession in Israel [27]. This is also partially attributable to the fact that to date, the profession has been characterized by constant change and development [38]. Additionally, counselors may take on different roles according to the needs of their specific school settings [38]. Many balance responsibilities as general members of the “educational team” [39], such as teaching classes, with systemic work supporting the school community [9,40]. These multiple expectations, including the demands placed on them by school administration and other partners, have been shown to have a substantial influence on counselors’ professional identity formation [1].

### 1.4. The School Counselor’s Role in Addressing CSA in Israel

Prevalence rates of sexual abuse among children in Israel have been as high as 18.7% in recent national findings [41,42,43]. Israeli counselors’ legal obligation to notify the authorities in such cases and the penalties for noncompliance are outlined in the Israeli Mandatory Reporting Act (1989). Also pertinent is the matter of peer-to-peer sexual assault, which can occur in school settings.

The school counselor’s professional responsibilities in these contexts are outlined in the Director General’s Circular [44,45] (guidelines on policy and procedure issued by the Ministry of Education) and the Professional Practice Standards in Educational Counseling. According to these sources, counselors must be notified of and respond to all reported incidents of sexual abuse, whether by meeting directly with the students concerned or guiding a teacher’s intervention [44,45]. Counselors further act as parental consultants and coordinate other aspects of the school’s educational response [9].

School counselors are expected to recruit partners in executing their professional obligations [9] and, as such, are accompanied in these efforts by a counseling supervisor and a district instructor of abuse prevention from the Ministry of Education’s psychological service (SHEFI). Both during interventions and afterward, these figures are available to consult and provide guidance [44].

### 1.5. School Counselors’ Coping When Addressing CSA in Israel

Recent studies examining Israeli educators’ experiences in coping with CSA among their students have highlighted their fear, loneliness, and traumatization [46]. Research on school principals has also indicated that educators’ varying coping strategies in response to CSA disclosure are influenced by both professional and personal experience [47] and cultural context. Studies show that educators utilize religious and cultural values in both their coping and intervention practices [8,46]; socio-cultural context has also been found to influence perceptions of abuse, reactions to disclosure, rates of reporting, and rates of support for child victims [48].

In Israel, differences between cultural subgroups significantly affect communities’ interventions in cases of CSA (e.g., [46,49,50,51]). Among the Israeli Jewish population, which constitutes approximately 74% of the total population, families often align with Western norms, featuring democratic relations and permissive parenting [48]. Conversely, Israeli Arab society’s traditional collectivism [52] tends to curb information disclosure to authorities [53,54].

Israeli Arabs account for 21% of the total population [55] and vary in religious affiliation (83.4% Muslim, 8.4% Christian, and 8.2% Druze), geographic location, and locality-based characteristics [56]. However, modernization has led to the gradual adoption of individualistic values and other changing social norms in Arab society as well [57,58]. At the same time, Arabs in Israel are further constrained in reporting by a sense of distrust and foreignness in their encounters with the state’s welfare and mental health care systems [54]. Nonetheless, both communities are subject to the same state and professional guidelines for CSA treatment and intervention.

Very limited studies have been conducted examining how school counselors in distinct socio-cultural contexts in Israel contend with CSA among their students. Researchers found that Druze counselors have been able to resolve cultural conflicts by abstaining from police intervention and referring families to therapy under the Israeli mandatory reporting system [59]. In another Israeli study, Arab school counselors reported significant difficulties with victim blaming and resistance to reporting among the parents of adolescents who had been sexually abused. Counselors have expressed frustration at being unable to provide professional help to their sexually abused students and describe feeling restricted due to their traditional cultural norms [60].

### 1.6. The Current Study

The current study was conducted as part of a larger project consisting of over 200 interviews with educators from diverse cultural and religious backgrounds (secular Jewish, religious Jewish, Arab Christian, Arab Muslim, Druze, and Bedouin) working in elementary schools in Israel. The purpose of the research project was to create a conceptual, multicultural framework for understanding the experiences of educational professionals coping with CSA among their students. The current study focused on the experiences of secular Jewish and Arab school counselors. The experiences of the other cultural groups included in the research project have been analyzed and addressed in the authors’ additional studies, from a multicultural perspective as well as community-specific analysis (e.g., [10,13,46,47,49,50,51,61]).

Previous studies have demonstrated school counselors’ critical role in CSA detection and prevention programs in schools [62,63]. Yet, a limited number have studied school counselors’ professional identity construction while coping with CSA cases, and more specifically, coping with those cases in different socio-cultural contexts. The purpose of this study is to fill this theoretical gap by obtaining the professional and socio-cultural viewpoints of school counselors regarding their complicated coping processes. To that end, the current study examines, from a comparative perspective, the experiences of Israeli Jewish and Arab school counselors coping with CSA disclosure. Considering the limited literature available, a qualitative approach was chosen, allowing for a detailed exploration of the meanings the counselors attribute to their experiences [64]. This study addresses the following questions: (1) How do Jewish and Arab school counselors in Israel cope with CSA disclosure among their students? (2) What processes of professional identity construction do the school counselors experience while coping with CSA disclosure among their students?

## 2. Method

### 2.1. Participants

A purposeful sampling strategy was used to identify and select individuals with particular knowledge of or experience as educators coping with CSA, the phenomenon of interest [65]. The sample [66] included 21 female Israeli school counselors, of whom 11 were Arab between the ages of 30 and 55 (M = 40.75, SD = 7.80) and 10 were secular Jewish between the ages of 32 and 58 (M = 46.60, SD = 8.11); all had 1 to 26 years of counseling experience in schools (Arab: M = 10.75, SD = 4.02; secular Jewish: M = 12.20, SD = 8.17). The researchers found no men engaged in the profession at all, reflecting its gendered characterization in Israel [40]. All participants worked in elementary schools at the time of the study and had significant experience coping with CSA as a part of their professional role. Most of the participants (17) worked in the north of Israel, with 2 in the center of the country and 2 in the Jerusalem region. Also, the participants were recruited using a snowball sampling method [67]. The sample size was chosen according to the principle of data saturation [68], widely referenced in thematic analysis research, indicating the point at which no new themes or codes ‘emerge’ from the data.

### 2.2. Measures

A semi-structured interview guide was used to avoid bias related to the researchers’ familiarity with the field of study. This guide was developed based on the literature and peer consultations and included the following subjects: coping with CSA disclosure (e.g., What would I see if I were able to watch from under a cloak of invisibility when a student exposed you, as a school counselor, to the matter of sexual abuse?); the perceived role of school counselors in CSA cases (e.g., What do you think the role of school counselors is in the context of the phenomenon of CSA?); and socio-cultural influences on coping with CSA (e.g., How does Arab/Jewish society refer to the phenomenon of child sexual abuse?).

### 2.3. Procedure

This study applied a qualitative approach, in order to achieve a multifaceted exploration of the meanings the participants ascribed to their experiences [64].

#### 2.3.1. Data Collection

The first author of this study conducted two open interviews and three semi-structured interviews. Sixteen additional semi-structured interviews were conducted by two M.Ed. graduate students in education and one MA student in social work, members of a multidisciplinary team involved in researching educators coping with CSA. The 21 semi-structured interviews were all based on the culturally-informed interview guide [69]. During the period of data collection, the researchers received continuous, dedicated training and supervision from expert researchers in the field, the fourth and fifth authors of this article, who led the multidisciplinary research group. The interviews were conducted as part of the students’ fulfillment of the requirements for a seminar or research thesis on the topic. Due to the coronavirus pandemic, nine interviews were conducted in person and twelve were conducted online (via Zoom). The interviews lasted 45–75 min and took place during the years 2019–2021, the data collection period of the larger project.

#### 2.3.2. Ethical Considerations

This study received approval from the Ethics Committees of the authors’ affiliated institutions. Privacy and confidentiality were ensured by restricting sensitive personal data to a locked file and utilizing pseudonyms and other such obfuscations in the interview transcripts. The participants all signed forms indicating their consent; moreover, consent was viewed not as a singular occurrence but as an ongoing matter, such that participants were encouraged to reject any question or ask to end the interview at any time they chose. Participants were reminded of these options in person and in writing at various points during the interview process [70]. Finally, before the interviews, the researchers apprised participants of the availability of professional assistance should the experience distress them in any way and supplied participants with the relevant contact information at the close of each interview.

### 2.4. Data Analysis

Five researchers were involved in this project, all experienced in the field of child maltreatment and qualitative research methods. To construct this study’s themes, two of the researchers began by repeatedly reviewing the transcripts using qualitative analysis software, Dedoose version 8.3.17, until the participants’ narratives were entirely familiar to them. Following this, the researchers engaged in a multistage process of both separately and jointly identifying recurring units of meaning [71]; synthesizing these into codes and code groups; isolating those that centrally defined the participant’s perceptions until all questions raised in the analysis were addressed and saturation was achieved [72]; checking these themes against the transcripts in order to further revise and expand on them; and formulating them into a conceptual model describing the meanings that the participants attributed to their experiences with CSA in their schools [72]. The analysis was at all stages fully inductive and grounded in the data, unbiased by preceding coding frames [68]. Peer debriefing was conducted throughout the analysis through meetings to discuss selected excerpts in order to reach a common agreement. In the findings, the themes were illustrated using representative excerpts from the participants, which were translated into English. The accuracy of this translation was corroborated by back-translating arbitrary selections into Hebrew.

Rigor and trustworthiness, which replace reliability and validity in qualitative studies [73], were essential concerns of the research and analysis process. Rigorous standards were promoted through several techniques: (1) The researchers performed investigator triangulation during the analysis, with some materials analyzed and coded by three of the five authors. (2) Through the whole research process, peer debriefing sessions were documented to discuss the source materials and interpretations. (3) To encourage reflection, the researchers kept detailed notes and a field diary. (4) To enhance their awareness of their impact on the research process, the researchers engaged in reflection sessions and team debriefing meetings. They also engaged in both formal and informal conversations with fellow team members and colleagues for this purpose.

The credibility of the findings is ensured through the inclusion of direct quotes from the participants, followed by an open examination of the researchers’ interpretations. For instance, the theme “Counselors’ professional identity transformation following the encounter with CSA among their students” is organized as (a) illustrative specifics of the participant; (b) an excerpt from his or her interview; and (c) a set of conclusions depicted in light of the data surrounding the participant’s experiences, which is synthesized with the overall themes. This method makes it possible for readers to ascertain whether the analysis is well-founded and to decide whether or not to endorse it [66,74,75]. As part of the verification process, the authors conferred with specialists in both CSA and qualitative research [76]. Finally, the researchers used member checking, reaching out to participants during data collection to refine or clarify points they had not previously considered [77].

## 3. Findings

The findings revealed the centrality of coping with CSA among their students (e.g., children’s inappropriate sexual behavior in/after school; sexual assault committed by a familiar person or a stranger; and incest) to the counselors’ professional development. Participants described their encounters with CSA disclosure as shocking but nevertheless as leading to a dynamic and reflective journey that shaped how they understood their professional role and responsibilities. Thus, from their narratives, two primary themes surfaced, reflecting the process of their professional identity construction: (1) Professional identity transformation following encounters with CSA disclosure, which deals with how the counselors redefine and refine their professional role following their coping with CSA cases; and (2) Integrating professional knowledge, attitudes, and engagement behaviors into the professional identity, referring to the ongoing transformation of their professional self-conception, aligning it with the norms and expectations of their chosen field.

### 3.1. Counselors’ Professional Identity Transformation following Encounters with CSA Disclosure

The counselors’ descriptions of their experiences in cases of CSA reflected a process of transition in their perception of professional roles. Early in their careers, their reactions were characterized by confusion, fear, disconnection, and stress; they lacked confidence as to how to properly respond and doubted their abilities. In some instances, counselors did not see themselves as accountable for managing such situations. However, both Arab and Jewish counselors depicted a trajectory of professional development in coping with CSA disclosure. Over time, as they gained experience, they came to accept the reality of CSA as a part of their professional role and their responsibility in intervention and prevention.

To begin with, the early years were described by some participants as an almost intrusive experience, where they lacked the ability to separate their work and private lives and to engage in operative thinking when experiencing intense emotions. As Mira, an Arab counselor with 24 years of seniority, related,

The first time I was exposed to sexual abuse, I felt sick. I kept to myself, feeling completely shocked; I couldn’t stand on my feet. I felt weak and shivery. I wavered many times about what I should and shouldn’t do. I felt detached from the world and even from my victimized student.

This counselor experienced such a severe physical reaction, it was as if she was paralyzed and unable to respond. There was also a sense of detachment, a common trauma response also shared by many victims. Other counselors similarly recounted secondary trauma symptoms, such as difficulty sleeping and troubling thoughts.

The severity of this shock may relate to the counselors’ expectations before beginning their careers. As Aliza, a Jewish school counselor with eight years of seniority, stated,

I came […] to be nice […] I brought sweets […] I didn’t expect this to happen here in the first week […] It confronted me with the reality of the job very, very quickly. It put it in perspective for me […] the dark side of the role […]

The full complexity of their profession may have been unclear to the counselors before they entered the workforce and even for the first few years of their careers. This lack of awareness may be linked to the powerful shock they experienced when they first encountered cases of CSA.

Noor, an Arab counselor with 20 years of seniority, described a corresponding but culturally specific challenge when facing CSA early in her career:

I used to deny the phenomenon… In my mind, this isn’t something that happens in our society. During my studies in educational counseling, I learned about it, but I didn’t care because I believed that there was no sexual abuse in our society.

When exposed to CSA, Noor resorted to denial and “splitting”, mentally banishing the painful phenomenon to cultures other than her own. While a common defense mechanism, this reflected the participant’s unpreparedness to address CSA in a professional capacity, even as she was being trained to do so.

However, both Jewish and Arab counselors also noted that their increased experience with CSA led eventually to a paradigm shift, leading them away from such thinking. Various emotional strategies were utilized to facilitate this transition to a more evolved professional identity: dealing with difficult emotions without splitting; learning to separate between their feelings and their thoughts; distinguishing between empathy and identification; and neutralizing values.

As the counselors became aware of the responsibilities of their position, they discarded their previous fears and dismissiveness and instead described themselves as having “a calling”. Amal, an Arab participant with 20 years of seniority, expressed the extent of this change:

I used to say: If I get stuck with sexual abuse cases, no way will I handle them, I’m not interested, it’s not my job. I don’t want to get involved in sexual abuse cases […] It’s not my role as a counselor… But today, I say it’s mine, and I won’t leave it for anyone else (to take care of) and won’t give up on any student.

Though in the past, she didn’t consider dealing with CSA part of her professional role, she later arrived at a determination to be exclusively responsible for every student.

Notably, the participants were, like many counselors, former teachers who were used to passing CSA cases on to others (including those who served as school counselors before them). Though their roles had changed, their coping methods initially remained similar to what they were previously. Yet, as they advanced their understanding of their new role, both Jewish and Arab counselors described a dramatic change in their coping ability. Einat, a Jewish counselor with 13 years of seniority, explained,

I think you develop some kind of defense mechanism […] so we’re able to keep on hearing those things. And we can keep hugging, we come home and continue to live, smile, and function. […] In the beginning, it crushed me, today I react totally differently. I’m more rational.

Substantial vulnerability was associated with CSA cases early on, as illustrated by this description of gaining the ability to protect oneself. Relationships and everyday functioning, adversely affected without this protection, were later saved by the ability to think rationally.

Having a sense of separation from the victim was another form of defense. As Amal, an Arab counselor with 20-year seniority, described,

It’s true that I’m empathic, but I don’t over-identify with the student. I’m in a more educated place to understand what I should and shouldn’t do. […] [T]oday I have more control over my feelings. I can be with the victim and ignore what happened to me (as a result of the case), I don’t let my thoughts take control of me. If they do, I won’t be able to help the children. So, I stop those feelings and thoughts and just stay with the victimized child.

This counselor distinguished “empathy”, feeling for the victim, from “identification”, or blurring the boundaries between their experiential worlds. Developing this separation mechanism shifted the focus from the counselor’s emotions to those of the child victim. This enabled her to be controlled and professional when involved in CSA cases. Further, accepting the limitations of her ability to help also improved the counselor’s professional response. Other participants also reported using self-talk to remind themselves that “the student is the victim, not my child” or that “the student needs me, I don’t need him”. By controlling their emotions, they were better able to focus on taking necessary action.

Some distinctive role-construction strategies were also identified among the Arab counselors only, relating to the gap between cultural values and role expectations. Mona, an Arab counselor with 20 years of seniority, explained,

I must neutralize myself from all the values I believe in my life, as I ought to be [on the side of] the student, treating him as a victim who has been entered into inappropriate sexual behavior for his age.

According to the counselor’s cultural values, sexual behavior was in a certain sense the victim’s responsibility; yet, she was expected as a school counselor not to victim-blame in cases of CSA. Hence, the counselor put aside her traditional values in order to focus on the victim’s needs.

As the theme highlights, the counselors’ descriptions of their experiences in CSA cases reflected a process of professional identity transformation. Confusion, fear, and lack of confidence characterized their reactions early in their career; however, using some emotional strategies, they were able to develop a sense of acceptance and resilience and view CSA as a normal part of their profession. Through experience, they developed a stronger sense of competence and self-efficacy in CSA cases.

### 3.2. Integrating Professional Knowledge, Attitudes, and Engagement Behaviors in the Transformative Professional Identity

After accepting the reality of CSA as part of their work, the counselors’ descriptions of their experiences in cases of CSA reflected a process of integrating knowledge of the profession, professional attitudes, and engagement behaviors into their new perception of their professional role. In recognizing their own intervention and prevention responsibilities, the school counselors felt more capable of recognizing and addressing the multiple fronts across which CSA affected the school system and providing comprehensive, personalized support to victims and their families. As a result of acquiring knowledge and skills, they were able to adopt a professional attitude and intervene effectively in both proactive and reactive ways.

Several Jewish and Arab counselors shared how acquiring knowledge through professional training gave them a sense of increased competence. Ahlam, an Arab counselor with 12 years of experience, shared,

I have decided within myself that I need to be strong, and that strength comes from awareness, learning, and participating in workshops and seminars about sexuality. […] Through lessons, courses, and workshops, I developed and excelled in my work. […] I’m more qualified now, and I have the knowledge and awareness.

Through training, Ahlam gained skills and knowledge she could apply to her role, making her more confident and capable and giving her the professional strength she needed.

Yet, both Arab and Jewish counselors argued that current training opportunities remained insufficient because they were largely elective and unstructured. Manar, an Arab counselor with five years of experience, also described the stigma associated with such materials:

Nobody talks sincerely about this subject. They are afraid to […] even read or discuss it […] although it’s of great importance in education. That’s why this year I decided to take a sexual education course. Sometimes I wonder whether I’m allowed to say what I’m studying. Will anyone understand me, or will they say: Isn’t there anything else to study? Those are […] barriers that restrict me.

Manar perceived a gap between her attitude and that of her community. However, she believed that as a counselor, it was her responsibility to study sexual education despite its taboo status. Implementing such an operational initiative for her own professional development required the courage to overcome cultural barriers.

Through professional training, the participants were able to provide more effective guidance and counseling services that responded appropriately to students’ needs. With their increased knowledge, counselors from both Arab and Jewish backgrounds gained a more refined ability to recognize CSA and understand its consequences as well as the importance of proper support for victims. This awareness formed the foundation of the overall professional attitude that guided their actions, decisions, and interactions as they sought to promote CSA intervention and prevention. Racheli, a Jewish counselor with 12 years of experience, described her understanding of her role as a case manager in instances of CSA requiring intervention:

We’re central to such cases. The whole intervention is on the counselor’s shoulders. She’s the one who leads the process […] manages the crisis. While she has partners, she has a big role to play in managing the event from the beginning to the end.

The school counselors’ professional attitude also included a commitment to ensuring victims received appropriate treatment and family support. They demonstrated an awareness of the need to help children feel safe and secure in order to begin the process of recovery, as well as a deep understanding of the importance of therapy to that process. Yael, a Jewish counselor with 12 years of experience, displayed this professional attitude when one victim’s parent could not provide the necessary support for his child:

The most important role that I play is to form a bridge between the victim student and the parent, ensuring the child is not left alone while supporting the parent’s ability to cope and support the child.

Yael understood that the child’s coping ability was strongly influenced by the parent’s ability to cope with the shocking news. She believed that the support she provided to parents would allow them to develop resilience and so would indirectly help the child as well.

This professionally grounded advocacy for therapy also arose in other circumstances where treatment was impeded. As Arab counselors with 14 and 5 years of experience, Noel and Miriam struggled with opposition to therapy derived from Arab traditional cultural norms, based on fears that it would threaten the marriageability of female CSA victims and even harm the whole family. Miriam explained,

Sexuality issues in our culture are immature and don’t understand the need for therapy for both victims and perpetrators. It’s all “fadiha (shame)” in our society, especially when the victim is a girl, thinking about how she’ll marry if she’s been sexually abused or has had therapy. According to her parents, she has no future, as they refuse to admit that she has been abused.

Noel reinforced this point and clarified the role of the school counselor in these circumstances:

As school counselors, we must continue to emphasize the benefits of therapy and trucking to mitigate the psychological and physical consequences of CSA. Our responsibility is to raise parental awareness about the need for therapy and support parents who need it. By standing up to society, assisted by social welfare services and the law, we must not give up.

The professional attitude cultivated by Noel and Miriam allowed them to recognize abuse’s psychological and cultural effects simultaneously. While acknowledging social norms, they firmly maintained an approach that met the needs of victimized students.

School counselors confronted CSA events both reactively and proactively through engagement behaviors. Reactive behaviors included seeking solutions to the different needs of everyone involved in incidents of CSA (students, parents, and teachers) while cooperating with other stakeholders (e.g., social welfare and psychological services). Proactive behaviors included designing and operating prevention plans, assisting both students and educational staff in recognizing and reporting signs of sexual abuse.

Mona, an Arab counselor with 12 years of experience, emphasized her reactive behaviors when describing her level of dedication as a school counselor:

Social welfare workers always tell me I’m a professional because I’m a very stubborn woman. I’m determined not to give up until the child receives therapy. Reporting child abuse to the police or sending the child to therapy isn’t enough for me. I’ll accompany him until his therapy is done. Professionals do reliable jobs, so if I turn to a welfare officer today, he knows he’s dealing with a professional.

In her capacity as a counselor—and reflecting her perception of this role—Mona accepted full responsibility from the beginning to the end, ensuring that all interventions were completed to the highest standard. She involved partners in charge of conducting therapy, but this did not diminish her own sense of professional obligation.

Ahlam, an Arab counselor with 12 years of experience, described her proactive behaviors:

The beginning of my responsibilities starts before sexual abuse happens, which means I need to “vaccinate” students to protect them and make them aware. Also, it’s our responsibility to guide teachers at the school on what to do in cases of sexual abuse and what to do to identify students who are sexually abused. My goal is to reach every student and every home and teach them what sexuality means.

With this use of the word “vaccine,” Ahlam emphasized the significance of her actions in preventing CSA. She saw it as paramount to promote healthy sex education, with the goal of averting inappropriate and harmful sexual behaviors. Her portrayal framed her role as valuable to children, teachers, and parents alike.

As the theme has highlighted, school counselors were more capable of recognizing multiple needs within their school system when dealing with CSA once they accepted CSA as part of their professional role. This enabled them to provide comprehensive, personalized support to victims and their families. Through acquiring knowledge and skills, they developed a professional attitude and effectively intervened in proactive and reactive ways.

## 4. Discussion

School counselors have been identified as playing a significant role in confronting CSA [7]; however, scant research has been conducted on the development of their professional identity in relation to this phenomenon. While previous studies have mainly focused on factors that influence reporting and identification patterns [62,63], this study qualitatively examined counselors’ perceptions regarding their professional identity construction process while coping with CSA among their pupils.

The current study portrayed how contending with CSA incidences acted as a catalyst in the participants’ journeys of professional growth. This development involved the transformative experience of moving beyond their previous roles (such as that of a teacher, a common initial career path for counselors in the Israeli context [40]), to embrace a new, distinct professional identity as a school counselor. Through self-reflection and exploration, the counselors articulated their professional values and beliefs and integrated their responsibilities regarding CSA into their professional personae. Achieving this integration and forming a coherent sense of identity within their counseling roles enabled them to function independently and may have served as a protective mechanism against burnout [20,78].

However, our findings revealed that the initial stages of this professional transformation were fraught with challenges that spanned the personal and professional realms. Past studies dealing with the experiences of school counselors contending with CSA have indicated that negative feelings are common, including depression, helplessness, and self-doubt [7]. In the present study, both the Jewish and Arab counselors showed a similar pattern of emotional upheaval after confronting the disclosure of CSA in the early stages of their careers. The experience of such overwhelming feelings undermined the fulfillment of their professional, legal, and ethical requirement to manage and regulate such events [45]. This sense of unpreparedness and lack of competence in managing CSA cases has been identified among Arab and Islamic professionals globally, including in the kingdoms of Saudi Arabia and Jordan [79,80], as well as among Arab educators within an Israeli context (e.g., [10]). The additional discovery made by the current study, which differs from these earlier findings, was the trend of outright denial of CSA among Arab educators in the early stages of their careers, driven by the belief that such incidents were impossible within their culture. A previous study on professionals in Saudi Arabia managing CSA cases similarly revealed a high degree of skepticism towards child disclosures, attributed to insufficient training on CSA and various cultural and religious factors [81,82]. Yet, the outright denial of the existence of CSA within their cultural context, as identified by the Arab educators in this study, represents a unique finding.

Alongside this, both Arab and Jewish school counselors viewed the challenge of CSA as integral to their professional and personal growth, crucial to their transition away from their role as teachers and the development of their identities as counselors. Their coping with CSA can be viewed as one of the “transformational tasks” that, as proposed by Gibson et al. [23], enables counselors to construct their role at each stage of their professional lives [23,83]. The developmental task of adjustment to expectations was, in particular, reflected in how the school counselors gradually balanced the idealized role they had imagined for themselves and the reality of professional expectations and restrictions [83]. As they gained experience, they also gained confidence regarding their role requirements in CSA situations, thus constructing “a personal definition of counseling” ([23], p. 21).

In internalizing their roles, the school counselors employed cognitive strategies to navigate the interplay between their personal and professional selves. First, by distinguishing between empathy and identification as an essential counseling practice [84], the counselors were able to prevent excessive emotional involvement and thus provide more child-focused, resilient, and balanced interventions. Arab counselors also notably utilized neutralization to reconcile their professional responsibilities with cultural values, actively promoting sex education and CSA awareness despite social norms such as “eb” (shame) and the prohibition against engaging with taboo subjects [53]. This was surprising in light of previous findings of Arab educators utilizing compromise to navigate their conflicting personal, cultural, and professional roles [13,47]. In fact, these counselors saw themselves as vehicles for change and accepted the consequences of their socially unacceptable acts.

Upon achieving a distinct identity through the recognition of CSA as part of their professional responsibilities, the counselors shifted their focus outward, actively seeking knowledge and collaborating with partners to address the needs of victims, their families, and the school community. In other words, they acted as educational leaders [85]. This process enhanced their capability for both reactive strategies [86]— focusing on managing stress from events that have already occurred (e.g., responding more adequately following CSA disclosure)—and proactive strategies [87], which are future-oriented approaches that view stressors as challenges and growth opportunities (e.g., providing sex education and prevention plans for students as well as education staff).

In addition, this study highlights the complex journey that Arab school counselors in Israel, as a minority group, navigate in constructing their professional identities while addressing the culturally taboo subject of CSA. Despite a gradual societal shift towards acknowledging CSA’s gravity and the importance of sex education [88,89], integrating these elements into their professional identity poses a substantial cultural challenge. Thus, consistent with prior research among Arab educators in Israel [13,46,50], Arab school counselors strive to strike a balance between acting as agents of social change and adhering to their cultural sensitivities. Yet, in instances where compromise is unattainable, they adopt a more proactive stance, positioning themselves as pivotal community figures driving change.

Past research on the cultural challenges faced by Arab professionals worldwide in managing CSA cases is limited and tends to focus on diverse professionals rather than specifically on educators. These studies primarily address reporting dilemmas and difficulties stemming from socio-cultural factors, such as fears of tarnishing the family’s reputation, community denial, the importance of a girl’s chastity in Arab Muslim culture, and the enduring stigma of sexual abuse [80,81,82]. The present study, along with other past qualitative research conducted by the authors in the Israeli context (e.g., [10,13,50,51,61]), echoes these findings in relation to Arab educators and further highlights the emotional consequences of the conflict between personal beliefs and professional responsibilities.

## 5. Limitations

As this is a qualitative study, the limited sample is designed to uncover unique characteristics for further exploration rather than to be statistically representative [90]. Given that the sample included elementary school and general education counselors only, further studies should address counselors with different role characteristics (e.g., in secondary or special education). Similarly, as the sample was comprised of women only, future studies could explore the effects of known gender differences in professional development [91].

On the other hand, the sample was heterogeneous in terms of both cultural context (it included both secular Jewish and Arab counselors from varied educational settings [92]) and years of seniority. Further research should more closely investigate cultural effects, e.g., the challenges associated with closed and/or religious communities. Regarding seniority, this factor was found to be significant in counselors’ professional identity development in general [83], a finding that was reflected in this study in relation to CSA in particular. Further research should examine the effect of seniority on the role construction process.

Finally, the findings of this study highlight the agency of both Arab and Jewish school counselors in dealing with CSA. Yet, this finding must be qualified by the fact that those school counselors already proactively involved in efforts to counter CSA were perhaps more likely to respond to our call for participation. Consequently, it is possible that school counselors with other perspectives were neglected in recruitment for the sample.

## 6. Implications for Theory, Research, and Practice

The findings of this study add to the existing literature on the professional identity construction of school counselors by clearly illustrating the role of dealing with CSA cases in this process. While it is known that school counselors’ development involves cultivating core values, moral principles, self-awareness, and self-knowledge [14], the current study pinpoints the stages of the process that are executed in the specific context of handling CSA. The findings emphasize an initial stage of significant changes to active perception, requiring the dynamic use of strategies and reflective thoughts and the presence of self-awareness and self-knowledge. Indeed, the second stage of professional functioning and coping using core values and moral principles is only possible after this transformational stage has occurred. Furthermore, the current study’s findings contribute to the literature by highlighting that the professional identity of school counselors is established not only in the social context [16] but also in the socio-cultural context as it pertains to CSA cases.

This study underscores the importance of training for school counselors and other professional staff that not only imparts professional knowledge about CSA but also encourages reflective practice through self-awareness and self-knowledge as vital for developing a solid professional identity [93]. Thus, counselor and student counseling training programs should integrate comprehensive CSA knowledge and foster reflection on both reactive and proactive strategies. Furthermore, framing the management of CSA as a professional growth opportunity can enhance counselors’ resilience and foster the development of proactive interventions that benefit schools, families, and communities.

The complexity of the professional identity construction of Arab school counselors requires an examination of legal and policy aspects. In their current form, professional and state guidelines for Israeli counselors in cases of CSA are tailored to Western socio-cultural and educational needs. Yet, as agents of change at school and in the community, key players in the development of school intervention policies, and key sources of guidance for teachers coping with CSA, it is imperative that school counselors receive culturally sensitive training on CSA intervention and prevention. Culturally sensitive updates to these documents are essential to ensure that Arab educators are receiving training that is relevant and useful for their particular challenges, and counselors should be made aware of the unique complexities associated with different cultural backgrounds.

The current study also found a significant difference between new and senior school counselors in their ability to cope both emotionally and professionally with the effects of CSA. Therefore, it is very important to focus on counselors who are in the initial stages of their careers in order to strengthen their resilience. This study shows that beyond acquiring professional skills and knowledge, counselors’ professional development is influenced by reflexive observation of their role-construction processes [94]. Field training should therefore focus on the professional processes that counselors go through throughout their career span and foster adapted tools for each developmental stage. For example, in the pre-disillusionment phase, when counselors experience mental and emotional overload, their skill in recruiting partners and support should be increased.

Lastly, our results indicate that more research is needed for separate, in-depth investigations of each cultural group. For example, a study focusing on Arab counselors will require a deeper exploration of the socio-political contexts that structure their experiences and perceptions and how these intersect with the process of constructing a professional identity. In addition, following the issues raised in this study, it would be worthwhile to conduct follow-up studies focusing on the interdisciplinary dynamics present in schools and how these may affect counselors’ dealings with CSA.

## Data Availability

Data are unavailable due to privacy restrictions.
